# Impact of the Internet on Medical Decisions of Chinese Adults: Longitudinal Data Analysis

**DOI:** 10.2196/18481

**Published:** 2020-09-03

**Authors:** Qianqian Ma, Dongxu Sun, Fangfang Cui, Yunkai Zhai, Jie Zhao, Xianying He, Jinming Shi, Jinghong Gao, Mingyuan Li, Wenjie Zhang

**Affiliations:** 1 National Engineering Laboratory for Internet Medical Systems and Applications The First Affiliated Hospital of Zhengzhou University Zhengzhou, Henan China; 2 National Telemedicine Center of China Zhengzhou, Henan China; 3 School of Management Engineering Zhengzhou University Zhengzhou, Henan China

**Keywords:** internet, medical decision, health care provider choice, adult, longitudinal data analysis, hierarchical medical policy

## Abstract

**Background:**

The internet has caused the explosive growth of medical information and has greatly improved the availability of medical knowledge. This makes the internet one of the main ways for residents to obtain medical information and knowledge before seeking medical treatment. However, little has been researched on how the internet affects medical decisions.

**Objective:**

The purpose of this study was to explore the associations between internet behaviors and medical decisions among Chinese adults aged 18 or over, including whether to go to the hospital and which level of medical institution to choose.

**Methods:**

With the adult residents (≥18 years old) in 12 regions including urban and rural areas taken as the research objects, the differences in medical choices of adults with various characteristics were analyzed, and generalized linear mixed models were adopted to analyze the longitudinal data of the China Health Nutrition Survey from 2006 to 2015.

**Results:**

Adult groups with different ages, genders, education levels, regions, places of residence, severities of illness and injury, years of suffering from hypertension, and history of chronic diseases showed diverse medical decisions, and the differences were statistically significant (*P*<.05). After controlling for these potential confounding factors and taking self-care as the reference, the probability of Chinese adults who participated in online browsing activities selecting hospital care was 0.82 (95% CI 0.69-0.98; *P*=.03) times that of residents who did not participate in online browsing activities. In terms of medical institution choices, adults who participated in online browsing activities were 1.86 (95% CI 1.35-2.58; *P*<.001) times more likely to opt for municipal medical treatment than primary care. However, the effect of online browsing on the selection probability of county-level hospitals was not significant compared with primary hospitals (*P*=.59). Robust analysis verified that accessing the internet had a similar effect on Chinese adults’ medical decisions.

**Conclusions:**

Chinese adults who use the internet are a little less likely to go to the hospital than self-care. The internet has broken down the barriers to obtain knowledge of common diseases and thus has a slight substitution effect of self-care on hospital care. Internet use may increase the probability of adults going to municipal hospitals. The rising tendency of visiting high-level medical institutions may be consequently exacerbated due to knowledge monopoly of severe and complicated diseases that is difficult to eliminate, and the increase in inconsistent and incomplete medical information online will blur the residents’ cognitive boundary of common diseases and severe diseases. Exploring the substantive impact of the internet on medical decision making is of great significance for further rational planning and utilization of the internet, in order to guide patients to appropriate medical institution.

## Introduction

Medical resources have been unequally distributed in different levels of hospitals in China for a long time, and this has resulted in the chaos of residents’ health-seeking behaviors [1,2]. According to statistics, the number of hospital beds in medical institutions in urban areas was 8.70 per 1000 people, compared with 4.56 per 1000 people in rural areas and 1.43 per 1000 people in township hospitals in 2018 [3]. Furthermore, the number of licensed doctors (assistants) per 1000 people in urban and rural areas was 10.91 and 4.63, respectively [3], which indicates the imbalance of medical resources in China. In other words, health resources have been allocated in big cities and superior hospitals. As a result, patients who suffer from acute or severe diseases, or those with stable or mild diseases, tend to select larger and professional hospitals rather than the smaller ones. The overcrowded high-level medical institutions and other medical institutions with insufficient patients reflect the inefficient utilization and wasting of health resources, and the chaos existing in medical practice [4]. In recent years, the hierarchical medical policy (HMP) has been proposed to steer patients from higher- to lower-cost providers [5]. The main idea behind the implementation of HMP is that the initial diagnosis is recommended to be carried out at the grassroots level, chronic and common diseases treated in the primary hospitals, and acute and intractable diseases referred to higher-level hospitals for treatment [6]. In 2018, the Chinese government proposed *Internet & health care*, a crucial development strategy which regards the internet as an important means to promote the implementation of HMP and optimize allocation of health care resources [7].

The period from 2006 to 2011 represents the initial development stage of China’s internet health care. Telemedicine gradually emerged in 2015, while online medicine and remote consultation were not widely used. However, patients or healthy people can obtain disease knowledge online through search engines such as *Baidu*, and professional medical websites such as *Good Doctor Online* and *Weiyi*. The statistical report on internet development in China has suggested that internet penetration has continued to grow and the popularity of the internet has gradually spread to the elderly from the young [8]. Given the convenience, high usability, and wider accessibility, internet has become a medium for the dissemination of health information, which has led to the inflated growth of online medical information and achieved the widespread sharing of medical knowledge. The internet provides us with a variety of health information, including drug information, basic definition and symptoms, treatment methods, and mental health information [9]. People browse health information through the internet to make further medical decisions. For example, patients who originally plan to go to a large hospital change their minds and choose home-based self-care or a nearby primary medical institution for treatment instead after browsing online, or they realize the seriousness of the disease from the internet and immediately seek treatment at a high-level medical institution.

Studies have shown that an increasing number of people tend to use the internet to obtain health care information [10-12], including older adults [13,14]. One study revealed that 57% of adults with acute coronary syndromes in 6 hospitals in Massachusetts and Georgia sought health information online [11]. Another survey found that 88% of participants with opioid treatment searched for information on medical topics online [10]. Furthermore, another study showed that 89% of 335 Chinese pregnant women who attended the antenatal clinic in a general hospital in Guangzhou used the internet to retrieve health information from the beginning of the pregnancy [15]. For Chinese patients with invasive breast cancer, the rate of internet information searching was reported to be 49% [16]. Previous studies have demonstrated that using the internet to obtain health information is very common in the internet era. Exploring the substantive impact of the internet on medical decision making is of great significance for further rational planning and use of the internet, to guide patients to the appropriate medical institution based on their illness.

However, there is limited evidence about how internet affects adult hospital choices. When it comes to the factors influencing residents’ choice of medical treatment, most previous studies have focused more on hospital-related factors, including hospital equipment, distance, time, cost, reputation, doctor level, etc [17-22]. A survey showed that the primary reasons for choosing private hospitals were the presence of a specialist, availability of good equipment and technology, and trust in treatment, whereas proximity, receiving enough information, and being well-treated were the reasons why participants chose family health centers in Samsun Province in Turkey [20]. A semistructured interview study involving 13 pregnant women in Denmark noted that the experience of pregnant women themselves or their peers and travel distance played a role in the women’s choice of delivery hospital [21], while Schuldt et al [22] believed that factors such as the distance to hospital, level of information about the treatment, number of respective treatments performed in the hospital per year, and complication rate had a significant impact on hospital choice. However, these studies have rarely involved internet use.

Several studies that involve internet use indicate that the relationship between the internet and medical decision making still needs to be clarified. Lee et al [23] pointed out that online health information had the potential to powerfully influence the health attitude and behaviors of a large proportion of the population, and affected the management of chronic diseases. However, an earlier study [24] suggested that the internet could enhance residents’ health-related knowledge and attitudes to a certain extent, but rarely changed their health-related behaviors. Similarly, in the study by Zwijnenberg et al [25], patients showed interest in online comparative health care information, but the impact of internet on patients’ decision making remained limited. Consequently, it is still unclear whether searching online information through the internet will affect patient’s decision to go to the hospital and the choice of health care providers.

Therefore, the purpose of this study was to explore residents’ decision-making behavior under the background of the internet era, and to analyze whether the internet could guide and channel patients to the suitable medical institution, so as to achieve hierarchical treatment. Based on measurements of longitudinal data from 2006 to 2015, the generalized linear mixed model was employed to explore the associations between internet use and medical decisions in general Chinese adult population, combined with other relevant factors influencing patients’ preference for hospital types.

## Methods

### Data Source

Data were extracted from the China Health and Nutrition Survey [26], an international cooperation project jointly conducted by the Carolina Population Center of University of North Carolina at Chapel Hill and National Institute of Nutrition and Food Safety of the Chinese Center for Disease Control and Prevention. The survey is a continuously open cohort with a multistage, stratified cluster random sampling method, covering 12 regions including Heilongjiang, Liaoning, Hunan, Shandong, Guizhou, Jiangsu, Guangxi, Hubei, Henan, Beijing, Shanghai, and Chongqing. These regions differ in geographical location, economic development, public resources, health conditions, and other demographic measures, making the survey informative, high-quality, nationally representative data. The entire data collection and collation process has been subject to good quality control. In addition, the desensitized and anonymous data have been publicly released online, without patient privacy.

Questions about internet behaviors have been set in the original Chinese questionnaire after 2006, such as the internet location, online browsing, online chat, online game playing, and the duration of internet behaviors. Thus, this paper selected 2006-2015 longitudinal data. Because the medical treatment of minors is often decided by the guardian rather than by the minors themselves, residents younger than 18 years were excluded, and the research object included only the adult group. After data cleaning, the records with missing key variables, such as health care–seeking behavior and internet use, were excluded. The final analysis included 10,164 records, of which there were 2032, 2280, 3145, and 2707 records in 2006, 2009, 2011, and 2015, respectively. Among them, 4877 records were obtained from the same individuals by repeated observations. A total of 7408 adult participants were involved in the analysis and 2121 participants had records that were repeated at least twice. Each participant was followed up for 1, 2, 3, or 4 times (ie, not everyone had 4 records), which suggests unbalanced longitudinal data.

### Internet Use and Health Care–Seeking Behaviors

The internet behavior was obtained through the questions “Do you participate in surfing the internet? (Yes/No)” and “Can you access the internet? (Yes/No)” on the questionnaire. Browsing online was chosen as a proxy variable for internet usage, because only individuals who participated in online browsing activities had the opportunity to access the internet medical information. At the same time, in order to test the robustness of the association between internet use and health care–seeking behaviors, internet access was used as another explanatory variable in the robustness analysis.

Health-seeking behaviors were obtained from the questions “What did you do when you felt ill?” and “Which medical institution did you seek first?” The patients made medical decisions in the following 2 steps: (1) Whether to go to the hospital and (2) Which hospital to go to. Therefore, the analysis of the impact of the internet on medical behavior was divided into 2 parts according to the decision-making process. First, did it affect the patient’s choice of whether to seek medical treatment, self-care, or hospital care? This was a 2-category event. Second, based on the level of medical institutions, we classified health care provider choices into primary-level hospital, county-level hospital, and municipal-level hospital, which was a 3-category event.

### Potential Confounders

Some other factors might influence residents’ decision to seek medical treatment. For example, people of different ages and genders show distinct preferences for hospitals. Geographical differences indicate diverse levels of modernization as well as economic and medical development, and thus residents’ choice of the hospital may be affected by the supply of local medical resources. Given the potential confounders, it is not enough to consider only the single-factor influence of internet behavior on medical decision making. Thus, the adjusted model included sociodemographic characteristics (marriage, age, gender, education), health supply (medical insurance, district, urban or rural), health needs (body mass index, severity of illness or injury, history of chronic illness, hypertension), and other factors as covariates to in-depth verify the influence of internet behavior. Details of the variables are presented in Table 1.

**Table 1:** Description of variables.

Variables and description		Variable assignment	
**Explained variable**			
	Medical choice		0=Self-care, 1=Hospital care
	Tier of hospital care		1=Primary hospital, 2=County hospital, 3=Municipal hospital
**Explanatory variables**			
	Online browsing		0=No, 1=Yes
	Internet access		0=No, 1=Yes
**Confounders**			
	Marital status		0=Married, 1=Other (single, widowed, divorced, or separated)
	Age		0=18-44 years old, 1=45-59 years old, 3=60-74 years old, 4=≥75 years old
	Gender		0=Female, 1=Male
	Education level^a^ (years)		Years of being educated
	Medical insurance		0=No, 1=Yes
	District		0=Center, 1=East, 2=West
	Residence site		0=Rural, 1=Urban
	Time		Survey year (1=2006, 2=2009, 3=2011, 4=2015)
	Disease/injury severity		1=Not severe, 2=Somewhat severe, 3=Quite severe
	Chronic diseases^a^		The number of chronic diseases diagnosed by doctors, including hypertension, diabetes, myocardial infarction, stroke, asthma, tumor
	BMI^a^ (kg/m^2^)		Body mass index, calculated by weight (kg)/height (m^2^)
	Hypertension (years)^a^		Years of suffering from hypertension

^a^Continuous variable.

### Statistical Analysis and Methodology

Data collation and cleaning were performed using RStudio 1.1.456 software (RStudio, Inc.). The random forest algorithm was applied to fill in the missing values of potential confounders (<10%) after removing duplicate records and missing samples of key variables. In descriptive statistical analysis, statistical charts and tables were adopted to analyze the changes in health care provider choices among Chinese adults, and the differences in health-seeking behaviors among adults with different characteristics. The quantitative data were described by mean and standard deviation, whereas qualitative data were analyzed using rate or composition ratio. Univariate analysis was performed by Wilcoxon rank-sum test, multisample Kruskal–Wallis rank-sum test, chi-square test, and Cochran–Mantel–Haenszel test. In multivariate analysis, because the data were longitudinal and the health care–seeking behaviors were characterized with 2 categories in the first step and 3 categories in the second step as the dependent variable, the mixed-effects binary or multinomial logit model, (ie, a generalized linear mixed model with binomial or multinomial distribution and logit link function) was perhaps the most appropriate statistical perspective for analyzing such data when accounting for the potential lack of independence in longitudinal data [27,28].

Methodologically, combining the strengths of both the generalized linear model and linear mixed model, the generalized linear mixed model extends the generalized linear model further to account for variation and correlation of longitudinal data. A random effect b_ik_ (i=1, 2, ..., m) was introduced and the logit link function was selected in the model. With *k*=0 serving as the reference, the model was expressed using the following equation [28]:

log(P_ijk_/P_ij0_)=X_ij_′β+b_ik_+ε_ijk_ k=1, ..., K **(1)**

where P_ijk_ denotes the probability that adult i makes a medical decision of k in survey year j, P_ijk_=Pr(Y_ij_=k|X_ij_); ε_ijk_ is the within-subject random error and was normally distributed as N(0,*σ*_ijk_^2^); b_ik_ is the between-subjects random effect on the *k*th logit component, and was assumed to be distributed as N(0,σ^2^_bk_); and X_ij_ is the covariate vector. The mixed-effects binomial logit model (K=1) and mixed-effects multinomial logit model (K=2) were established by GLIMMIX Proc Step in SAS software, version 9.4 (SAS Institute Inc.) [29]. All tests were two-sided at the significance level α=.05 and *P*<.05 indicated statistical significance.

## Results

### Health Care Provider Choices for Chinese Adult Residents

**Figure 1:**
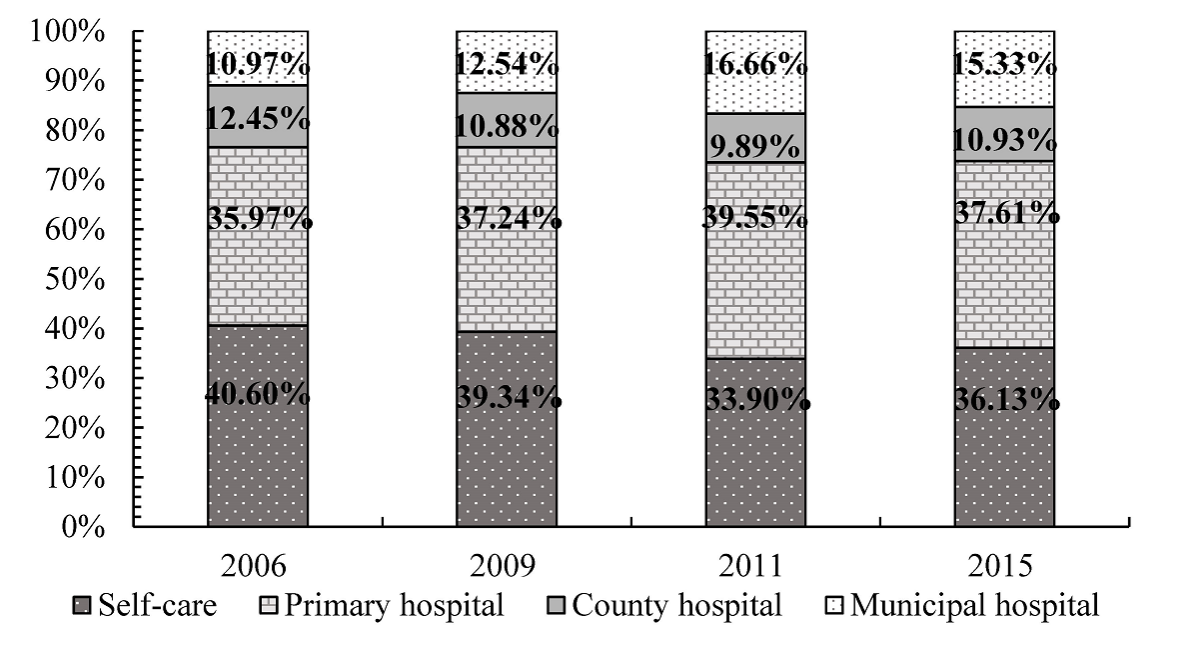
Health care provider choices of Chinese adult residents from 2006 to 2015.

On the whole, primary care and self-care were the main medical treatment choices for Chinese adults after they were sick or injured, accounting for 37.80% (3842/10,164) and 37.05% (3766/10,164) of the total records, respectively, followed by municipal and county hospitals. From 2006 to 2015, the proportion of consultations at primary medical institutions increased by 1.64% (from 35.97% to 37.61%), which indicated the moderate effects of HMP. The proportion of residents choosing municipal hospitals grew by 4.36% (from 10.97% to 15.33%), whereas the figures for choosing self-care and county hospitals both decreased (Figure 1).

### Factors Influencing Adults’ Medical Decisions

Taking the data in 2015 as an example, people with different characteristics had various preferences for health care (Tables 2 and 3).

First, for self-care versus hospital care choice, there was a significant correlation between adults’ age and their medical choices (ie, as they grew older, more patients chose to go to the hospital for treatment instead of self-treatment; χ^2^_3_=63.0, *P*<.001). The factor of disease or injury severity was also found to be statistically significantly associated with medical choices. Patients with more severe illness or injury were more likely to choose hospital care (χ^2^_2_=94.3, *P*<.001). In addition, education levels (*P*=.005), residence sites (*P*<.001), years of suffering from hypertension (*P*<.001), and history of chronic diseases (*P*<.001) differed significantly between those who chose self-care and those who chose hospital care (α=.05). However, gender, marital status, medical insurance, and BMI were not significantly associated with the choice of self-care or hospital care in univariate analysis (Table 2).

Second, for the tier of hospital care in Table 3, adult groups with different genders, education levels, regions, places of residence, severities of illness and injury, years of suffering from hypertension, and history of chronic diseases showed diverse choices of medical institutions, and the differences were statistically significant (*P*<.05). For instance, those with higher education mainly selected municipal hospitals (χ^2^_2_=76.1, *P*<.001). The proportions of urban residents’ choices of hospital were ranked as primary hospitals, municipal hospitals, county-level hospitals, whereas rural residents’ choices of hospitals were ranked as primary hospitals, county hospitals, municipal hospitals (χ^2^_2_=159.7, *P*<.001).

**Table 2:** Medical choices for people with different characteristics in 2015 (N=2707).

Variables		Medical choice			Hypothetical test		
		Self-care (N=978)	Hospital care (N=1729)		χ^2^ (*df*)/W	*P* value	
**Age (years), n (%)**				63.0 (3)		<.001	
	18-44	232 (23.72)	226 (13.07)				
	45-59	320 (32.72)	528 (30.54)				
	60-74	323 (33.03)	725 (41.93)				
	≥75	103 (10.53)	250 (14.46)				
**Gender, n (%)**					2.4 (1)	.12	
	Male	449 (45.91)	739 (42.74)				
	Female	529 (54.09)	990 (57.26)				
**Marriage status, n (%)**				2.0 (1)		.16	
	Married	836 (85.48)	1441 (83.34)				
	Others	142 (14.52)	288 (16.66)				
Education level (years), mean (SD)		8.140 (4.48)	7.57 (4.53)		899,572^a^	.005	
**Region, n (%)**					4.3 (2)	.11	
	East	388 (39.67)	737 (42.63)				
	Center	323 (33.03)	506 (29.27)				
	West	267 (27.30)	486 (28.11)				
**Residence site, n (%)**					13.8 (1)	<.001	
	Urban	518 (52.97)	786 (45.46)				
	Rural	460 (47.03)	943 (54.54)				
**Medical insurance, n** **(%)**					0.01 (1)	.92	
	No	36 (3.68)	61 (3.53)				
	Yes	942 (96.32)	1668 (96.47)				
**Disease or injury severity, n (%)**					94.3 (2)	<.001	
	Not severe	487 (49.80)	548 (31.69)				
	Somewhat severe	444 (45.40)	1005 (58.13)				
	Quite severe	47 (4.81)	176 (10.18)				
Hypertension (years), mean (SD)		2.01 (5.38)	4.13 (8.16)		717,065^a^	<.001	
BMI (kg/m^2^), mean (SD)		24.15 (3.84)	24.31 (3.78)		821,957^a^	.23	
Chronic diseases, mean (SD)		0.34 (0.62)	0.62 (0.80)		685,490^a^	<.001	

^a^Wilcoxon rank-sum test.

**Table 3:** Hospital choices for people with different characteristics in 2015 (N=1729).

Variables		Tier of hospital care			Hypothetical test	
		Primary hospital (N=1018)	County hospital (N=296)	Municipal hospital (N=415)	χ^2^ (*df*)	*P* value
**Age (years), n (%)**					10.2 (6)	.12
	18-44	137 (13.46)	37 (12.50)	52 (12.53)		
	45-59	293 (28.78)	106 (35.81)	129 (31.08)		
	60-74	451 (44.30)	111 (37.50)	163 (39.28)		
	≥75	137 (13.46)	42 (14.19)	71 (17.11)		
**Gender, n (%)**					6.4 (2)	.04
	Male	415 (40.77)	145 (48.99)	179 (43.13)		
	Female	603 (59.23)	151 (51.01)	236 (56.87)		
**Marriage status, n (%)**					4.2 (2)	.12
	Married	836 (82.12)	258 (87.16)	347 (83.61)		
	Others	182 (17.88)	38 (12.84)	68 (16.39)		
Education level (years), mean (SD)		6.87 (4.47)	7.84 (4.45)	9.12 (4.35)	76.1 (2)^a^	<.001
**Region, n (%)**					11.9 (4)	.02
	East	401 (39.39)	136 (45.95)	200 (48.19)		
	Center	307 (30.16)	85 (28.72)	114 (27.47)		
	West	310 (30.45)	75 (25.34)	101 (24.34)		
**Residence site, n (%)**					159.7 (2)	<.001
	Urban	413 (40.57)	79 (26.69)	294 (70.84)		
	Rural	605 (59.43)	217 (73.31)	121 (29.16)		
**Medical insurance, n** **(%)**					3.7 (2)	.16
	No	41 (4.03)	5 (1.69)	15 (3.61)		
	Yes	977 (95.97)	291 (98.31)	400 (96.39)		
**Disease or injury severity, n (%)**					30.7 (4)	<.001
	Not severe	371 (36.44)	78 (26.35)	99 (23.86)		
	Somewhat severe	563 (55.30)	179 (60.47)	263 (63.37)		
	Quite severe	84 (8.25)	39 (13.18)	53 (12.77)		
Hypertension (years), mean (SD)		3.67 (7.74)	3.71 (7.05)	5.58 (9.63)	13.9 (2)^a^	<.001
BMI (kg/m^2^), mean (SD)		24.26 (3.72)	24.77 (4.20)	24.12 (3.61)	4.0 (2)^a^	.13
Chronic diseases, mean (SD)		0.54 (0.73)	0.65 (0.85)	0.79 (0.89)	26.3 (2)^a^	<.001

^a^Multisample Kruskal–Wallis rank-sum test.

### Relationship Between Internet Use and Medical Decisions

Adults who did not browse the internet presented an obvious preference for primary hospitals, supplemented by self-diagnosis and treatment. By contrast, people who browsed the internet had different medical treatment–seeking behaviors, and they preferred self-care, followed by medical care from primary hospitals and municipal hospitals (Table 4). With the time (year) as a stratified variable, it was found that the use of the internet was significantly related to the choice of health care provider among adults after controlling the time variable by the Cochran–Mantel–Haenszel test (χ^2^_3_=170.4, *P*<.001).

**Table 4:** Association between internet use and medical decisions in different survey years.

Year: Internet use		Self-care, n (%)	Primary hospital, n (%)	County hospital, n (%)	Municipal hospital, n (%)
**2006: Online browsing**					
	Yes (N=124)	65 (52.42)	20 (16.13)	16 (12.90)	23 (18.55)
	No (N=1908)	760 (39.83)	711 (37.26)	237 (12.42)	200 (10.48)
**2009: Online browsing**					
	Yes (N=225)	129 (57.33)	41 (18.22)	14 (6.22)	41 (18.22)
	No (N=2055)	768 (37.37)	808 (39.32)	234 (11.39)	245 (11.92)
**2011: Online browsing**					
	Yes (N=502)	207 (41.24)	128 (25.50)	42 (8.37)	125 (24.90)
	No (N=2643)	859 (32.50)	1116 (42.22)	269 (10.18)	399 (15.10)
**2015: Online browsing**					
	Yes (N=441)	205 (46.49)	117 (26.53)	39 (8.84)	80 (18.14)
	No (N=2266)	773 (34.11)	901 (39.76)	257 (11.34)	335 (14.78)

#### Impact of the Internet on Choosing Self-Care Versus Hospital Care

Taking self-care as the reference group, the mixed-effects binomial logit model was employed to analyze whether online browsing would influence patient’s decision to visit hospital. Based on the univariate analysis of Model 1, Models 2 and 3 further introduced different confounders that potentially affect patients’ medical decision to validate whether the relationship between online browsing and patient decisions was still significant. Models 1-3 all clarified that Chinese adults who participated in online browsing activities were less likely to go to the hospital than those who did not participate in online browsing activities. As revealed in Model 3, the odds ratio was 0.82 (*e*^–0.20^; 95% CI 0.69-0.98; *P*=.03) in the group that participated in online browsing activities compared with those that did not participate in online browsing activities (Table 5).

**Table 5:** Results of a generalized linear mixed-effects binomial logit model analyzing the influence of internet use on choosing self-care versus hospital care.

Effects		Model 1 (unadjusted model)		Model 2		Model 3	
		Coefficient (95% CI)	*P* value	Coefficient (95% CI)	*P* value	Coefficient (95% CI)	*P* value
**Fixed effects**							
	Intercept	0.70 (0.64 to 0.76)	<.001	0.72 (0.53 to 0.92)	<.001	0.55 (0.15 to 0.95)	.007
**Online browsing (ref=No)**							
	Yes	–0.52 (–0.66 to 0.38)	<.001	–0.24 (–0.41 to –0.08)	.004	–0.20 (–0.37 to –0.02)	.03
**Age (ref=18-44)**							
	45-59			0.16 (0.02 to 0.30)	.03	–0.01 (–0.16 to 0.14)	.91
	60-74			0.21 (0.06 to 0.37)	.008	–0.09 (–0.26 to 0.07)	.27
	≥75			0.34 (0.13 to 0.55)	.001	–0.05 (–0.28 to 0.17)	.64
**Gender (ref=Female)**							
	Male			–0.05 (–0.16 to 0.06)	.40	–0.07 (–0.19 to 0.04)	.23
**Time (ref=2006)**							
	2009			0.07 (–0.07 to 0.20)	.32	0.07 (–0.08 to 0.22)	.36
	2011			0.38 (0.25 to 0.51)	<.001	0.33 (0.18 to 0.48)	<.001
	2015			0.29 (0.16 to 0.43)	<.001	0.25 (0.10 to 0.40)	.001
**Region (ref=Center)**							
	East			0.04 (–0.08 to 0.17)	.49	–0.02 (–0.15 to 0.11)	.76
	West			0.04 (–0.10 to 0.18)	.55	0.02 (–0.12 to 0.17)	.76
**Residence site (ref=Rural)**							
	Urban			–0.60 (–0.71 to –0.49)	<.001	–0.71 (–0.82 to –0.60)	<.001
**Marriage status (ref=Married)**							
	Others			–0.15 (–0.29 to –0.01)	.04	–0.15 (–0.30 to 0.003)	.046
Education level				–0.02 (–0.03 to 0.00)	.01	–0.01 (–0.03 to 0.001)	.08
**Disease/injury severity** **(ref=** **Not severe** **)**							
	Somewhat severe					0.85 (0.74 to 0.95)	<.001
	Quite severe					1.49 (1.29 to 1.68)	<.001
Chronic diseases						0.32 (0.22 to 0.42)	<.001
Hypertension						0.01 (–0.001 to 0.02)	.09
**Medical insurance (ref=** **No** **)**							
	Yes					0.03 (–0.13 to 0.19)	.73
BMI						–0.02 (–0.03 to 0.004)	.15
**Random effect**							
	Intercept, variance	2.40	<.001	2.47	<.001	2.82	<.001

#### Impact of the Internet on the Choices of Tier of Hospital Care

**Figure 2:**
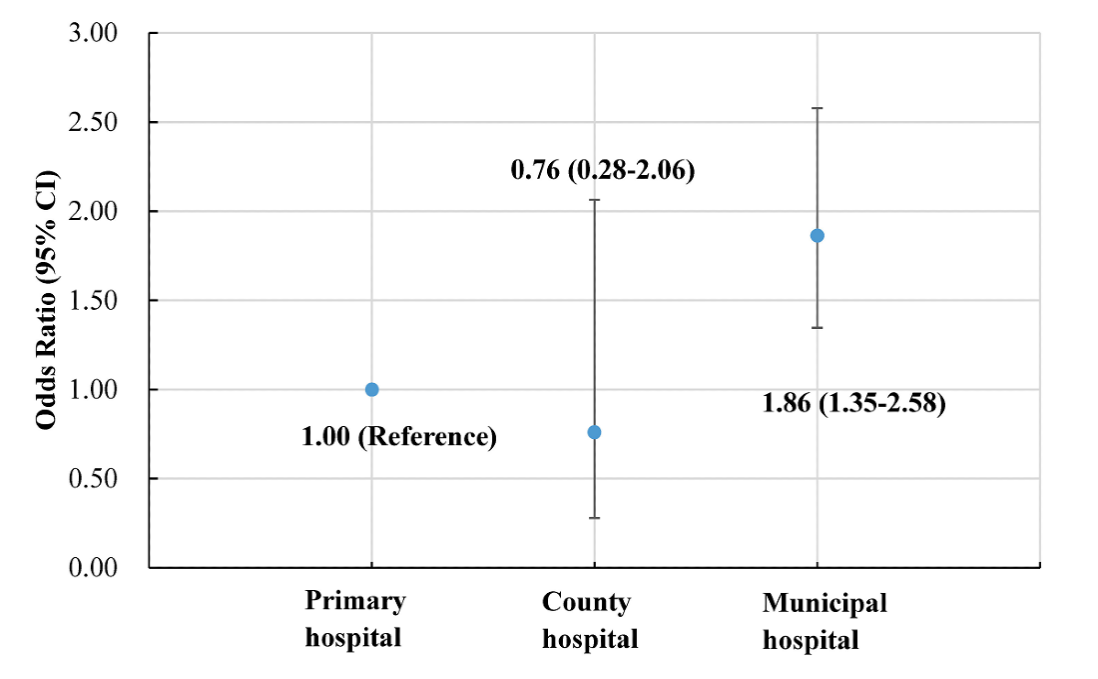
Odds ratio estimates based on Model 6.

Taking the primary medical institution as the reference group, 3 mixed-effects multinomial logit models (Models 4-6) were established by using different factors that might affect hospital choices as control variables. All parameter estimates of the models were shown in Multimedia Appendix 1, and the key results we were most interested in are presented in Table 6. The result showed that Chinese adults who participated in online browsing activities were more likely to choose municipal hospitals than primary medical institutions, whether in the unadjusted analysis (Model 4) or in the models adjusted for confounding factors (Models 5 and 6). The multifactor Model 6 hinted that after controlling for as many confounding factors as possible, residents participating in online browsing activities were 1.86 (*e*^0.62^; 95% CI 1.35-2.58; *P*<.001) times more likely to opt for municipal medical treatment than those who did not participate in online browsing activities (Figure 2). However, the effect of online browsing on the selection probability of county-level hospitals was not significant compared with primary hospitals (*P*=.59).

**Table 6:** Results of generalized linear mixed-effects multinomial logit model analyzing the influence of online browsing on medical provider choice (ref=primary hospital).

Model and dependent variable		Online browsing		
		Coefficient	(95% CI)	*P* value
**4^a^**				
	County hospital	–0.05	(–0.97 to 0.86)	.90
	Municipal hospital	1.15	(0.51 to 1.78)	<.001
**5^b^**				
	County hospital	–0.31	(–1.28 to 0.66)	.53
	Municipal hospital	0.51	(0.20 to 0.81)	.001
**6^c^**				
	County hospital	–0.27	(–1.27 to 0.73)	.59
	Municipal hospital	0.62	(0.30 to 0.95)	<.001

^a^Only explanatory variable was included in the model.

^b^The confounders included in the model were the same as those in Model 2.

^c^The confounders included in the model were the same as those in Model 3.

#### Robust Analysis

Tables 7 and 8 present the results of robust analysis using “internet access” as another explanatory variable instead of the existing online browsing. As revealed in Model 9, the odds ratio was 0.85 (*e*^–0.16^; 95% CI 0.74-0.99, *P*=.03) in the group that could access the internet compared with that which could not access the internet. Model 12 showed that compared with primary hospital, the probability of residents who could access the internet selecting municipal hospital was 1.57 (*e*^0.45^; 95% CI 1.20-2.07, *P*=.001) times that of residents who did not access the internet. Besides, there was no preference gap for primary and county hospitals (*P*=.98). Robust analysis verified similar results that the internet had a certain effect on adults’ medical choices.

**Table 7:** Results of analyzing the influence of “accessing the internet” on medical choice behaviors (self-care versus hospital care, ref=self-care).

Model	Internet access		
	Coefficient	(95% CI)	*P* value
7^a^	–0.45	(–0.57 to –0.34)	<.001
8^b^	–0.18	(–0.32 to –0.04)	.01
9^c^	–0.16	(–0.31 to –0.01)	.03

^a^Only explanatory variable was included in the model.

^b^The confounders included in the model were the same as those in Model 2.

^c^The confounders included in the model were the same as those in Model 3.

**Table 8:** Results of analyzing the influence of “accessing the internet” on the choice of hospital (ref=primary hospital).

Model and dependent variable		Internet access		
		Coefficient	(95% CI)	*P* value
**10^a^**				
	County hospital	0.16	–0.52 to 0.84	.64
	Municipal hospital	0.81	0.27 to 1.35	.003
**11^b^**				
	County hospital	–0.03	–0.78 to 0.71	.93
	Municipal hospital	0.35	0.09 to 0.60	.008
**12^c^**				
	County hospital	0.01	–0.76 to 0.78	.98
	Municipal hospital	0.45	0.18 to 0.73	.001

^a^Only explanatory variable was included in the model.

^b^The confounders included in the model were the same as those in Model 2.

^c^The confounders included in the model were the same as those in Model 3.

## Discussion

### Principal Findings

Based on longitudinal data from 2006 to 2015, this paper analyzed the impact of internet on the medical decisions among Chinese adults through generalized linear mixed models. The results showed that the internet had a certain effect on adults’ medical decisions. First, regarding the impact of whether to go to the hospital, adults with internet behaviors (eg, browsing information online, accessing the internet) were less likely to go to the hospital. Patients tended to self-care, which presented a partial substitutive effect of self-diagnosis and treatment on hospital care. Second, in terms of hospital selection, compared with primary hospitals, the use of the internet might not change the probability of choosing county hospitals, but it might increase the probability of going to municipal hospitals for advanced treatment. The study has theoretical and practical implications on how to regulate internet health care and guide patients to seek medical institutions, and has a reference to the promotion and application of internet medical treatment.

Chinese adults with internet behaviors are more likely to self-diagnose and treat at home than visiting hospitals, which is consistent with some research descriptions [30,31]. Yang et al [30] pointed out that in the “internet +” era, online medical platforms provided an effective way to alleviate the high demand for hospitals. As the popularity of the internet has increased dramatically among people, browsing and selecting health information have become a basic approach before determining whether to visit hospitals further [31]. A study of 164 perinatal women in Korea showed that some women, who sought informal medical help online, would be more likely to change their medical decisions only according to internet information, without consulting doctors (*P*<.001) [32]. Concerning the reasons for choosing self-care instead of primary care, some studies have given explanations [10,33,34]. One study noted that almost half of health information searchers (48%) reported that health information online could help them take better care of themselves, and two-thirds of adults (67%) showed increasing awareness of health issues through internet [33]. Turan et al [34] and others suggested that online access to reliable disease information could abate anxiety, boost the feelings of self-efficacy, and reduce the use of medical services. The popularity of the internet can effectively overcome traditional obstacles and achieve easy access to health information for prevention and treatment [10]. All in all, the internet can break down the barriers to the knowledge of common diseases, reduce the asymmetry of information between patients and doctors to some extent, and improve patients’ awareness and access to basic health knowledge, thereby reducing the possibility of using medical services.

By contrast, this study found that the internet might exacerbate the tendency of going to higher-level medical institutions for medical treatment. The information browsed on the internet is not able to resolve the monopoly of knowledge about intractable and severe diseases. In addition, residents’ misunderstanding of medical expertise can cause health anxiety, for instance, misinterpretation of physical symptoms as signs of serious diseases, accompanied by persistent fear of serious illness [35]. Some studies have reported that internet health information searchers were more likely to have health concerns than nonseekers, and adult seekers tended to rate their health status as poor [36,37]. Furthermore, a random effect meta-analysis demonstrated that online health information seeking was positively correlated with health anxiety (*r*=0.34, 95% CI 0.20-0.48, *P*<.001) [37]. At the same time, given the privacy principles, the medical information that can be retrieved is often partial, subjective, and even biased, which aggravates the limitations and incompleteness of residents’ awareness of the disease. In a semistructured interview on the use of Chinese language internet information on cancer, most of the 20 respondents reported that they encountered internet health information with questionable quality [38]. An observational study showed that some sites provide harmful information, and the proportion of these sites was much higher than sites providing reliable information on cancer treatment (N=247) [39]. The studies above hint at the reasons why the use of internet might increase the probability of residents going to high-level medical institutions.

Unlike previous studies that have paid more attention to the impact of hospital-related factors on patients’ medical decision making, our study focused on internet use. Especially in the internet era, as mentioned previously, the internet has played a vital role in residents’ decision making on their choice of hospitals [40]. Li et al [41] demonstrated that there was a strong association between online health communities information and patient decisions of switching from online to offline medical services. One study suggested an association between online health information–seeking behaviors and some health behaviors, such as physical activity, fruit and vegetable consumption, alcohol use, and hypertension medication adherence [23]. However, there are few studies that deal with such health behaviors as whether to go to a hospital and what level of hospital to choose, under the influence of the internet. This research has innovatively analyzed the influence of internet behavior on medical choices by following the 2 steps of the decision-making process. In addition, some factors such as age or education level might be associated with medical decisions. According to a survey in Samsun Province in Turkey, patients aged 18 years or younger and 65 years or older preferred family health centers, whereas those aged 19-64 presented a higher preference for private hospitals [20]. In addition, it was pointed out that the level of education affected patients’ choices [20]. Our study not only explored the impact of the internet use on the residents’ choice of health care provider by univariate analysis, but also deeply took other confounding factors into account, including age, gender, region, urban or rural, education, disease severity, chronic medical history, and BMI, that might affect health care choices from the perspective of residents.

### Limitations

This study has employed the generalized linear mixed models to delve into the associations between internet use and medical decisions with longitudinal data, which fills in the gaps of current related research and provides a reference for policy makers. To our knowledge, this is the first time that the mixed-effects multinomial logit regression, an appropriate method for processing longitudinally correlated multiclass data [42,43], is adopted for modeling medical institution choices in China. However, there are some limitations in this study. First, variables such as occupation, income, transportation mode, self-perceived life happiness index, and internet browsing time were not included in the model as confounding factors due to high percentage of missing data. In addition, when interpreting the results, only the internet behaviors in the main forms of “online browsing” and “having access to the internet” were considered, rather than interactive internet medical behaviors, such as online consultation with doctors. As a result, further study focusing more on medical information can be conducted with an in-depth assessment of network usage, including network usage time, languages of online health information (in English or in Chinese) [36], content of information (Western medicine or traditional Chinese medicine), level of trust in online information, etc, which can deeply portray the impact of the internet on residents’ health care–seeking behaviors.

### Conclusions

With the advent of the internet, the availability of health care information has improved. The internet has become a pivotal source of medical information for Chinese residents [13,44]. This study has found that compared with self-care, internet use slightly reduces the probability of patients going to the hospital to some extent. In addition, compared with primary hospitals, the internet seems not to change the probability of choosing county hospitals, although it may increase the probability of adults going to municipal hospitals for high-level health care. The internet has broken down the barriers to the knowledge of common diseases, shortened the gaps in health information accessibility, and has produced a slight substitution effect of self-diagnosis and treatment on hospital care. However, the knowledge monopoly of difficult and complicated diseases cannot be eliminated, and at the same time, the increase in inconsistent, incomplete, and commercialized medical information has also brought noise to decision making, and will blur the residents’ cognitive boundary of common diseases and severe diseases. Consequently, the rising tendency of visiting high-level medical institutions may be exacerbated, which is unable to guide patients to hierarchical diagnosis and treatment. It is necessary to further regulate the normativeness of medical-related websites, ensure the correctness and scientificity of medical knowledge online, and reduce the noise of medical information correspondingly in order to achieve the standardized dissemination of medical knowledge. For example, it is recommended to promote the implementation of telemedicine and internet hospitals, and make it an important means to support health self-management and rehabilitation with extensive application of internet technology, and guide patients to make medical decisions, which will ultimately contribute to the formation of hierarchical diagnosis and treatment order.

## Appendix

Multimedia Appendix 1 Estimation results of generalized linear mixed-effects multinomial logit models analyzing the influence of online browsing on medical provider choice.

## Abbreviations

CHNS: China Health and Nutrition Survey
HMP: hierarchical medical policy
