# Supplementary material for: Impact of the Internet on Medical Decisions of Chinese Adults: Longitudinal Data Analysis
Source: J Med Internet Res. 2020 Sep 3;22(9):e18481. doi: 10.2196/18481 (PMC7499166; doi:10.2196/18481)
Supplement: Multimedia Appendix 1 [file jmir_v22i9e18481_app1.pdf]

Effects	**Model 4 （unadjusted model）**				**Model 5**				**Model 6**			
	County hospital		Municipal hospital		County hospital		Municipal hospital		County hospital		Municipal hospital	
	Coefficient (95%CI)	*P*	Coefficient (95%CI)	*P*	Coefficient (95%CI)	*P*	Coefficient (95%CI)	*P*	Coefficient (95%CI)	*P*	Coefficient (95%CI)	*P*
**Fixed effects**												
Intercept	-9.38 (-9.82,-8.93)	<.001	-9.13 (-9.55,-8.72)	<.001	-9.82 (-10.82,-8.82)	<.001	-3.68 (-4.23,-3.13)	<.001	-12.72 (-14.75,-10.68)	<.001	-4.39 (-5.34,-3.45)	<.001
**Browse online(ref=No)**												
Yes	**-0.05 (-0.97,0.86)**	**.90**	**1.15 (0.51,1.78)**	<.001	**-0.31 (-1.28,0.66)**	**.53**	**0.51 (0.20,0.81)**	**.001**	**-0.27 (-1.27,0.73)**	**.59**	**0.62 (0.30,0.95)**	<.001
**Age(ref=18~44)**												
45~59					0.02 (-0.69,0.72)	.96	0.50 (0.23,0.77)	<.001	-0.14 (-0.88,0.59)	.70	0.35 (0.06,0.63)	.02
60~74					0.41 (-0.36,1.17)	.30	0.85 (0.56,1.15)	<.001	0.17 (-0.65,0.98)	.69	0.59 (0.27,0.91)	<.001
≥75					0.47 (-0.60,1.54)	.39	1.57 (1.18,1.96)	<.001	0.21 (-0.91,1.34)	.71	1.25 (0.82,1.68)	<.001
**Gender(ref=Female)**												
Male					-0.21 (-0.79,0.37)	.48	-0.18 (-0.37,0.02)	.07	-0.22 (-0.82,0.38)	.47	-0.22 (-0.42,-0.02)	.03
**Time(ref=2006)**												
2009					-0.06 (-0.61,0.49)	.83	0.03 (-0.24,0.31)	.82	-0.29 (-0.92,0.33)	.36	0.09 (-0.23,0.41)	.58
2011					-0.75 (-1.32,-0.17)	.01	-0.20 (-0.46,0.07)	.15	-1.06 （-1.70,-0.42）	.001	-0.22 (-0.53,0.08)	.16
2015					-0.52 (-1.13,0.08)	.09	-0.36 (-0.63,-0.08)	.01	-0.86 (-1.54,-0.18)	.01	-0.34 （-0.67,-0.02）	.04
**Region(ref=Center)**												
East					0.43 (-0.26,1.11)	.22	-0.05 (-0.27,0.17)	.67	0.26 (-0.45,0.97)	.48	-0.12 (-0.36,0.12)	.34
West					0.17 (-0.57,0.91)	.65	-0.25 (-0.49,0.00)	.045	0.27 (-0.50,1.04)	.48	-0.26 (-0.53,0.00)	.054

**Residence site(ref=Rural)**												
Urban					-0.64 (-1.30,0.03)	.06	1.94 (1.64,2.24)	<.001	-0.75 (-1.44,-0.07)	.03	1.98 (1.62,2.36)	<.001
**Marriage status(ref=Married)**												
Others					-0.61 (-1.44,0.23)	.15	-0.22 (-0.47,0.03)	.09	-0.57 (-1.43,0.29)	.19	-0.24 (-0.51,0.03)	.08
**Education level**					0.13 (0.05,0.20)	<.001	0.13 (0.11,0.16)	<.001	0.15 (0.07,0.22)	<.001	0.15 (0.12,0.18)	<.001
**Disease/injury severity（ref= Not severe）**												
Somewhat severe									0.76 (0.23,1.29)	.01	0.69 (0.46,0.92)	<.001
Quite severe									2.12 (1.43,2.81)	<.001	2.09 (1.67,2.51)	<.001
**Chronic diseases**									0.09 (-0.29,0.48)	.63	0.34 (0.18,0.50)	<.001
**Hypertension**									0.02 (-0.02,0.05)	.42	0.00 (-0.01,0.01)	.81
**Medical insurance(ref=No)**												
Yes									0.44 (-0.30,1.19)	.24	-0.09 (-0.41,0.23)	.58
**BMI**									0.08 (0.02,0.15)	.01	0.00 (-0.03,0.03)	.97
**Random effect**												
Intercept(Variance)	404.87	<.001	404.60	<.001	374.27	<.001	1.93	<.001	380.10	<.001	2.35	<.001
